# PENCALC: A program for penetrance estimation in autosomal dominant diseases

**DOI:** 10.1590/S1415-47572010005000054

**Published:** 2010-09-01

**Authors:** Andréa R. V. Russo Horimoto, Márcio T. Onodera, Paulo A. Otto

**Affiliations:** Departamento de Genética e Biologia Evolutiva Instituto de Biociências, Universidade de São Paulo, São Paulo, SPBrazil

**Keywords:** penetrance (rate, value), computer program, maximum likelihood estimation

## Abstract

We present a computer program developed for estimating penetrance rates in autosomal dominant diseases by means of family kinship and phenotype information contained within the pedigrees. The program also determines the exact 95% credibility interval for the penetrance estimate. Both executable (PenCalc for Windows) and web versions (PenCalcWeb) of the software are available. The web version enables further calculations, such as heterozygosity probabilities and assessment of offspring risks for all individuals in the pedigrees. Both programs can be accessed and down-loaded freely at the home-page address http://www.ib.usp.br/~otto/software.htm.

Accurate penetrance estimates are important for determining genetic disease recurrence risks in families where incompletely penetrant Mendelian disorders are segregating or for establishing genetic map locations by linkage analysis. While crude penetrance estimates can be rapidly derived by dividing the number of observed individuals expressing a disease phenotype by a rough estimate of the probable number of carriers in a given pedigree exhibiting autosomal dominant inheritance, deriving exact maximum likelihood estimates of carrier status at an individual level is time consuming and tedious. In this paper, we describe the structure and use of a computer program designed to be user friendly and assist genetic counselors and gene mappers to make accurate penetrance estimates in all sizes and complexities of autosomal dominant pedigrees, including those containing consanguineous loops and twin pairs.

The program deals with the situation of a single monogenic locus (**A, a**), with allele **A** dominant in relation to allele **a**. In the case of human autosomal dominant diseases, the homozygous condition **AA** is generally either unknown or very rare (given a low population frequency of the pathogenic allele **A**), so that in pedigrees with cases of autosomal dominant disease the affected individuals are almost invariably **Aa** heterozygotes, while normal individuals are either **aa** homozygotes or non-penetrant **Aa** heterozygotes. In this sense, the penetrance rate **K** is assumed to be the probability of an **Aa** heterozygote being affected: K = Prob(affected|Aa).

The penetrance rate estimation can be performed for polymorphic traits from familial aggregates including pairs of twins or other groups of close relatives, or, in the case of rare human diseases, through the analysis of phenotype segregation in pedigrees (a complete revision on this subject can be found in Horimoto and Otto, 2008). The algorithms are based on methods detailed by Rogatko (1986) and Horimoto (2009).

PenCalc for Windows was developed using Microsoft Visual Basic 6.0. This compressed, self-installing program can be obtained free of charge from the home page http://www.ib.usp.br/~otto/software.htm. The same page contains the access link to the PenCalcWeb Internet (www) program, developed using Active Server Pages (ASP), through the languages VBScript and Jscript. Both penetrance programs are the intellectual property of the authors, and as such, any use of or reference to the materials included in them, must contain an explicit reference to their origin. Feedback from users is welcome and will be used to improve the program and to correct unforeseen flaws.

Both programs are described in figures obtained directly in real time from screen images generated by the programs themselves.

First we describe PenCalc for Windows. To illustrate the operation of the program, we will use as example the hypothetical pedigree shown in Figure 1. At left of this figure is represented the whole pedigree and at right the filtered genealogy from which we identify the following tree structures pertinent to penetrance estimation: three affected (penetrant) individuals (II-4, III-4, and IV-1); four obligate non-penetrant individuals (I-1, II-1, II-3, and III-1); two normal individuals without offspring descending from an obligate (penetrant or non-penetrant) carrier of the gene (II-5 and II-6); and two 2-generation trees of normal individuals, one with two individuals in the second generation (II-2, III-2, and III-3), the other with three second-generation individuals (III-5, IV-2 to IV-4).

Options **File**, **Data Input** and **Help** appear in the main menu of the program's opening page. The **File** menu accesses options for exiting the program, for saving or printing the text file generated by the program to show the likelihood function, the estimate of K (penetrance value) and its exact 95% credibility interval. The **Data Input** menu program allows the writing of a data file through the **File** submenu option **Create** (Figure 2), accessing an existing data file through the **File** submenu option **Open**, or entering the data through specific program forms (**Screen** option, Figures 3 and 7). The **Help** menu accesses a graphic interface with the program credits (option **About...**) or gives information on the use of the user's **Manual** in pdf format.

The input of consanguineous structures is far more complicated; we shall use the example shown in Figure 5, where individuals I-1 and II-2 are assumed to be both descendents of related obligate carriers. In order to simplify not only the calculations but also the input of data, it is assumed that the dominant allele has been transmitted to the individual IV-2 of the last generation by either individual I-1 or II-2: the tree is then split into two different configurations (1 and 2), and their corresponding data entered separately.

**Figure 1:**
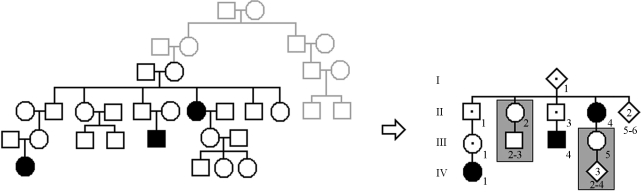
Hypothetical pedigree with individuals affected by an autosomal dominant disease. The whole pedigree is shown on the left; on the right all individuals and tree structures pertinent to penetrance estimation are shown – the gray boxes indicate the two trees of normal individuals that occur in the filtered pedigree.

**Figure 2:**
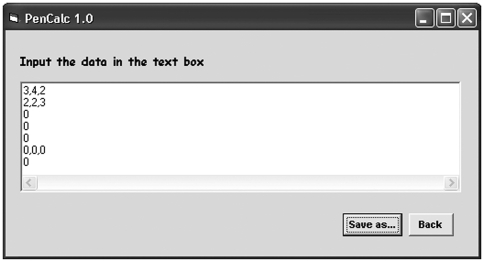
Interface for pedigree data input. Information on the pedigree structure under study is typed into the text box in a standardized manner using commas to separate items on the same line and carriage return to terminate lines (for full details, refer to the pdf manual). The data on the first line **(3,4,2)** indicates the number of affected penetrant individuals, obligate non-penetrant individuals and normal individuals without offspring descending from obligate carriers (penetrant or non-penetrant), respectively. In the second line **(2,2,3)**, the first figure **(2)** is the number of 2-generation trees of normal individuals; the digits that follow **(2,3)** are their corresponding offspring numbers. The next three lines containing one zero indicate, respectively, that no trees exist of normal individuals with 3, 4, or 5 generations; the line with three zeros, that there are no same-sex twin-pairs with both individuals normal, or one normal and one affected, or both affected; the zero on the last line, that no consanguineous trees occurred in the pedigree. The **Back** command button returns the main screen without saving the input data. The **Save As** button uses Windows standard commands for saving the data. When this takes place, the program automatically exhibits the results on the graphic interface (see Figure 8).

**Figure 3:**
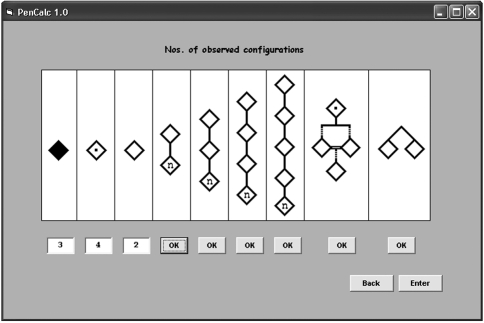
Graphic interface for data input through specific program forms, accessed through the **Data Input** submenu option **Screen**. The three leftmost text boxes were filled out, respectively, with the numbers of affected penetrant, obligate non-penetrant, and normal individuals without offspring descending from obligate carriers of the gene. The **OK** command buttons access the screens for data input of 2-, 3-, 4-, and 5-generation trees of normal individuals descended from obligate carriers (Figure 4), trees with consanguineous unions (Figure 6), and twin-pair occurrences (Figure 7); the **Back** command button returns to the main screen, without saving the input data, and the **Enter** command button returns to the main screen and the results are exhibited.

**Figure 4:**
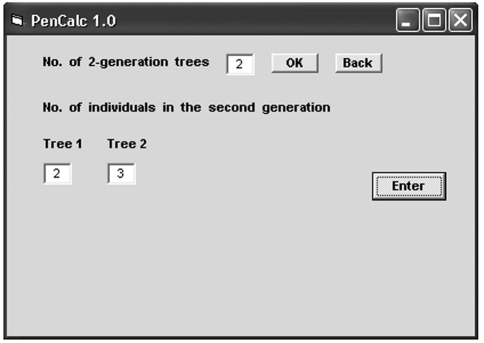
Final aspect of the graphic interface for data input for 2-generation trees of normal individuals, after the number **(2)** of two-generation trees of normal individuals is entered and the **OK** command button is keyed in. The **Enter** button saves the data in the boxes labeled Tree 1 and Tree 2 and then returns to the initial screen of data input (Figure 3). Similar graphic interfaces are presented for data input of 3-, 4-, and 5-generation trees of normal individuals (details on data input for these cases are explained in the program manual).

**Figure 5:**
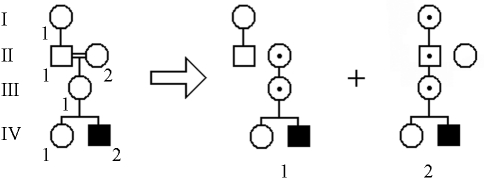
Example of a consanguineous tree (at left) that is split into two different configurations, 1 and 2. Configuration 1 has the following structures: one two-generation tree of normal individuals (I-1 and II-1), two non-penetrant obligate carriers (II-2 and III-1), one affected (penetrant) individual (IV-2) and one normal individual without offspring (IV-1), born to an obligate carrier. Configuration 2 has the following structures: three obligate non-penetrant carriers (I-1, II-1, and III-1), one affected (penetrant) individual (IV-2), and two normal individuals without offspring (II-2 and IV-1), both born to obligate carriers. All these data should be entered into the input window for consanguineous data shown in Figure 6.

**Figure 6:**
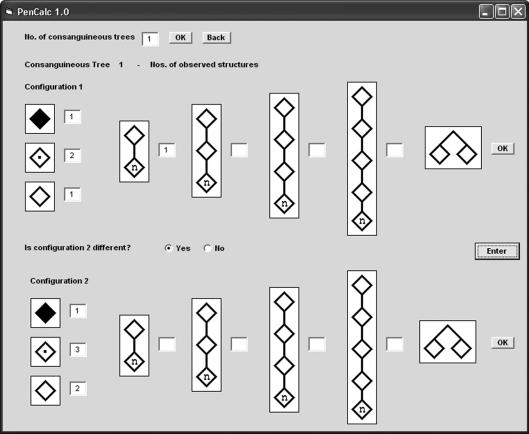
Input of consanguineous trees (maximum number: 2). When no consanguineous trees occur (which is the case of the example pedigree in Figure 1), there is no need to access this data input window. The window shown here is using the data derived from the example in Figure 5. In the case of configuration 1 of Figure 5 there exists one two-generation tree of normal individuals and when the option is chosen the program automatically opens another window for the input of the number (n) of individuals in the last generation (that is 1 in the worked example). Other details for entering the data from trees with a more complex structure (as well as for other additional procedures) are described in the program manual.

We next describe the program PenCalcWeb. Initially, the program shows graphic interfaces for entering data, similar to those for PenCalc for Windows. Because of the flexibility of the programming language, PenCalcWeb is far more intuitive to use than PenCalc for Windows. Only the final screen with results is shown in Figure 10.

**Figure 7:**
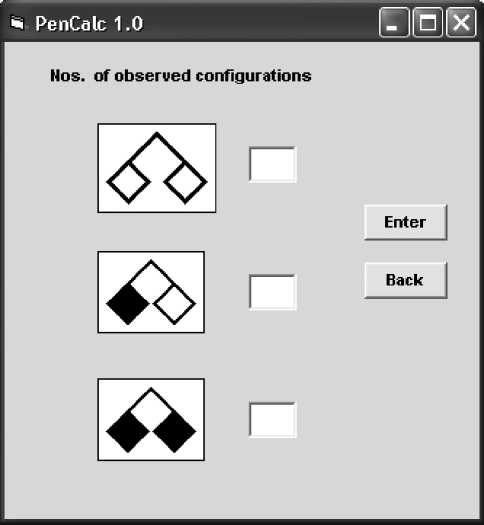
Data input of possible types of twin-pair occurrences (both normal individuals; one normal individual and one affected; both affected). These structures did not occur in the example pedigree of Figure 1 and the data input window is shown here just for descriptive purposes. The window above is accessed through data input windows shown in Figures 3 and 6 (respectively for non-consanguineous and consanguineous trees).

**Figure 8:**
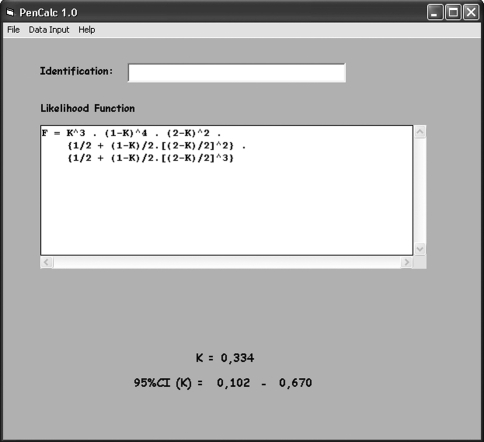
Interface showing the final results derived from the example pedigree given in Figure 1. After all the pertinent data are keyed in, the program exhibits the estimate of the penetrance rate K, its exact 95% credibility interval, and (inside the text box) the formula for the corresponding likelihood function. A blank field with the option to identify the pedigree is also presented by the interface. The results can be recorded or printed through the **File** submenus options **Save as** or **Print**. The **File** submenu option **Exit** closes the program, erasing all data that were not saved.

**Figure 9:**
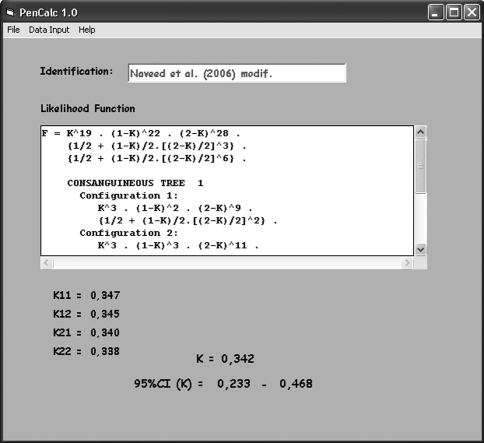
Interface of the program with the results for a pedigree containing two consanguineous trees (example adapted from Naveed *et al.*, 2006). The final likelihood function of the family consists of a common non-inbred part multiplied by each one of the four possible combinations of the formulae of one split tree from the first consanguineous tree and one from the other. The final estimate of K for the whole pedigree is obtained by weighing each of these four estimates K11, K12, K21, and K22 by the corresponding reciprocals of their variances. The lower and upper limits of the 95% credibility interval of the final estimate are also obtained by weighing the individual lower and upper limits of each Kij estimate by the corresponding reciprocals of the variances var(Kij). In the case of the worked example of Figures 5 and 6, there is just one consanguineous tree and the final K estimate is obtained by combining the separate estimates for each of the two possible configurations.

**Figure 10:**
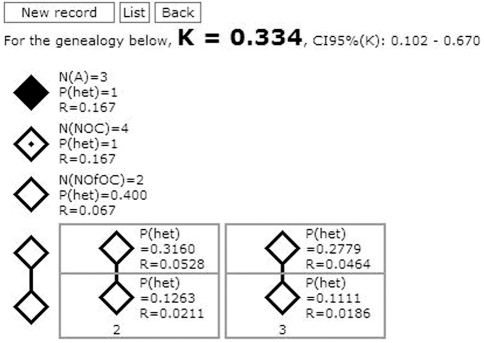
Graphic interface presenting (a) the structures corresponding to the components of the same example pedigree used in the description of PenCalc for Windows; (b) the penetrance value and its respective exact 95% credibility interval; (c) the heterozygosity probability and the offspring risk for all individuals inside the filtered pedigree.
